# Yushida: an IoT-based smart injection system for long-term management of chronic diseases

**DOI:** 10.3389/fdgth.2026.1900971

**Published:** 2026-08-07

**Authors:** Lifang Lei, Renjun Ma, Fang Lin, Lingjie Yang, Jin Peng, Yurou Yang, Yalin Yin, Meihua Yang

**Affiliations:** 1 Xiamen Amoytop Biotech Co., Ltd, Xiamen, Fujian, China; 2 Fujian Provincial Medical Products Administration, Xiamen, Fujian, China

**Keywords:** adherence monitoring, chronic disease management, digital health, internet of things, smart injection system, treatment adherence

## Abstract

In the management of chronic diseases requiring long-term subcutaneous injections (such as pediatric short stature and chronic viral hepatitis), dosing accuracy and treatment adherence are critical factors affecting therapeutic efficacy. Pediatric populations, in particular, present unique physiological and behavioral challenges that demand stringent safety, precise dose accuracy, and robust adherence monitoring from injection devices. However, existing injection devices still exhibit notable limitations in tracking medication behavior, assessing adherence, and managing long-term treatment. This paper introduces Yushida (Xiamen Amoytop Biotech Co., Ltd.), an Internet of Things-based smart injection system, incorporating a concealed-needle cartridge, a sensing system, Bluetooth connectivity, voice guidance, and automatic data logging. The article systematically examines the device architecture and digital functionalities of the device and compares them with representative smart injection systems currently available. The potential clinical utility of the system and the challenges associated with its broader implementation are also discussed. Although quantitative evidence directly evaluating the clinical impact of Yushida remains limited, several prospective clinical and real-world studies are currently underway and are expected to further clarify its clinical utility. By providing a balanced assessment of current technologies and evidence, this paper seeks to offer an objective perspective on the future development of digitalized injection therapies.

## 1 Introduction

In the long-term management of chronic diseases, precise, safe, and convenient drug delivery is fundamental to ensuring therapeutic efficacy and patient quality of life. This is particularly prominent in clinical areas requiring prolonged and regular injection regimens, such as pediatric short stature [including growth hormone deficiency (GHD), idiopathic short stature, and Turner syndrome] and viral hepatitis (1–3). The therapeutic protocols for these conditions not only necessitate rigorous dosing accuracy and timely administration but also require continuous monitoring of physiological responses and treatment adherence to ensure optimal clinical outcomes (4–7). Especially for pediatric and adolescent patients, who are in critical stages of growth and development, the demands for safety, operational ease, and pain management are even higher, hence posing more stringent requirements on the design and performance of injection devices (8–10).

Currently, conventional needles and syringes are widely used for drug delivery. However, they present significant limitations. Exposed needles often cause significant pain and injection anxiety, while complex manual steps not only affect the injection experience but may also lead to dosing inaccuracies or adverse injection-site reactions due to improper handling. Furthermore, some drugs require reconstitution prior to use, which makes the process cumbersome and time-consuming (11–13).

To address these challenges, various delivery devices have been developed over time. Prefilled syringes, pre-loaded with medication or equipped with integrated mixing systems for drugs and diluents, have simplified the administration process and enhanced dosing accuracy; their compact design also facilitates portability and storage (14–17). Subsequently, the pen-type syringe (injection pen) further simplified the storage and administration of multi-dose medications, enabling more patients, particularly children, to complete the administration independently (18). These injection pens typically execute injections via a button mechanism, eliminating the need to manually push a plunger (19, 20). Despite these advancements in convenience and precision, the therapeutic process still relies heavily on subjective recording and execution by patients or caregivers. This phenomenon often leads to data omissions and delayed feedback, which in turn hinders the objective assessment of efficacy and influences long-term treatment confidence. Consequently, there is an urgent clinical need for a novel injection tool that integrates precision injection, an enhanced comfort experience, data monitoring, and remote intelligent management to bridge the gap from simple drug administration to comprehensive, long-term digital management.

Yushida, a registered trademark of Xiamen Amoytop Biotech Co., Ltd. (Registration No.: Min-Xie-Zhu-Zhun 20252140035), is an Internet of Things (IoT)-enabled smart injection pen launched in China in 2025. It is currently utilized for the subcutaneous administration of long-acting growth hormone, Pegpesen (Inpegsomatropin; Xiamen Amoytop Biotech Co., Ltd.), and long-acting interferon, Pegbing® (Peginterferon α-2b; Xiamen Amoytop Biotech Co., Ltd.) (21, 22). Pegpesen is indicated for the treatment of GHD in pediatric patients aged three years and older (22, 23). Pegbing® is used for the treatment of chronic hepatitis B (CHB) in adults, as well as in combination with nucleos(t)ide analogues to achieve sustained clearance of hepatitis B surface antigen in adult CHB patients, thereby facilitating the attainment of a clinical cure (24, 25). Both medications use a Y-shape polyethylene glycol (PEG) modification technology (23, 26), with their once-weekly administration, thus providing a favorable opportunity for the integration of smart devices. Built upon this foundation, Yushida combines device connectivity, intelligent guidance, and automated data recording within a digital injection-management platform. The system is designed to support treatment monitoring and information sharing during long-term therapy by linking patients, caregivers, and healthcare professionals. The above features may offer potential advantages for managing chronic diseases, particularly in pediatric populations, although their impact on adherence and clinical outcomes remains to be established through further study.

Driven by these clinical and implementation needs, this technology-focused article describes the design, system architecture, workflow, intelligent functions, and potential clinical-management applications of the Yushida smart injection system. We further compare the device with representative commercially available smart injection systems and discuss its distinguishing features within the context of current connected drug delivery technologies. In addition to objectively discussing potential strengths, we critically examine the system's limitations and the challenges associated with its broader implementation and clinical adoption. Furthermore, in alignment with current digital healthcare trends, we also discuss the future development trends of intelligent drug delivery systems, with the aim of providing new insights and references for the precise treatment management of chronic diseases.

## 2 Literature search strategy

We searched PubMed, Scopus, and Web of Science from inception to June 2026 for literature on IoT-based drug delivery, connected injectors, digital therapeutics, and chronic care. Search terms included combinations of “smart injection system”, “connected injector”, “drug delivery device”, “IoT healthcare”, “digital health”, “treatment adherence”, “chronic disease management”, and related terms. Additional references were identified through manual screening of cited literature. Additionally, the official websites of the FDA, EMA, and NMPA/CMDE were searched for relevant injector regulatory guidance documents. Inclusion criteria encompassed: a: original research, reviews, regulatory guidance, and technical reports related to injection pens, auto-injectors, and connected injection devices; b: publications relevant to chronic disease management, treatment adherence, and digital health. Conference abstracts lacking peer review, press releases, and promotional materials were excluded. We paid attention to literature describing connected injection technologies, adherence-monitoring systems, pediatric injectable therapies, and digital health solutions relevant to long-term treatment, particularly. Then, publications were selected based on their relevance to device design, usability, connectivity, adherence monitoring, clinical applications, and implementation challenges rather than through predefined inclusion criteria.

## 3 System architecture and functional design of Yushida

### 3.1 Device architecture

The smart injection system adopts a modular design with integrated hardware and software. It consists of a reusable electronic injection pen (including the pen body, display screen, buttons, injection actuation mechanism, control circuit, and lithium battery) and a mobile digital management platform (Patent Nos. CN119770797A and CN119770798A). Notably, the device is designed to be used with separately supplied pre-filled medication cartridges and injection needles. To minimize the risk of contamination and maintain drug sterility, no components of the device come into direct contact with the therapeutic agent.

**Figure 1:**
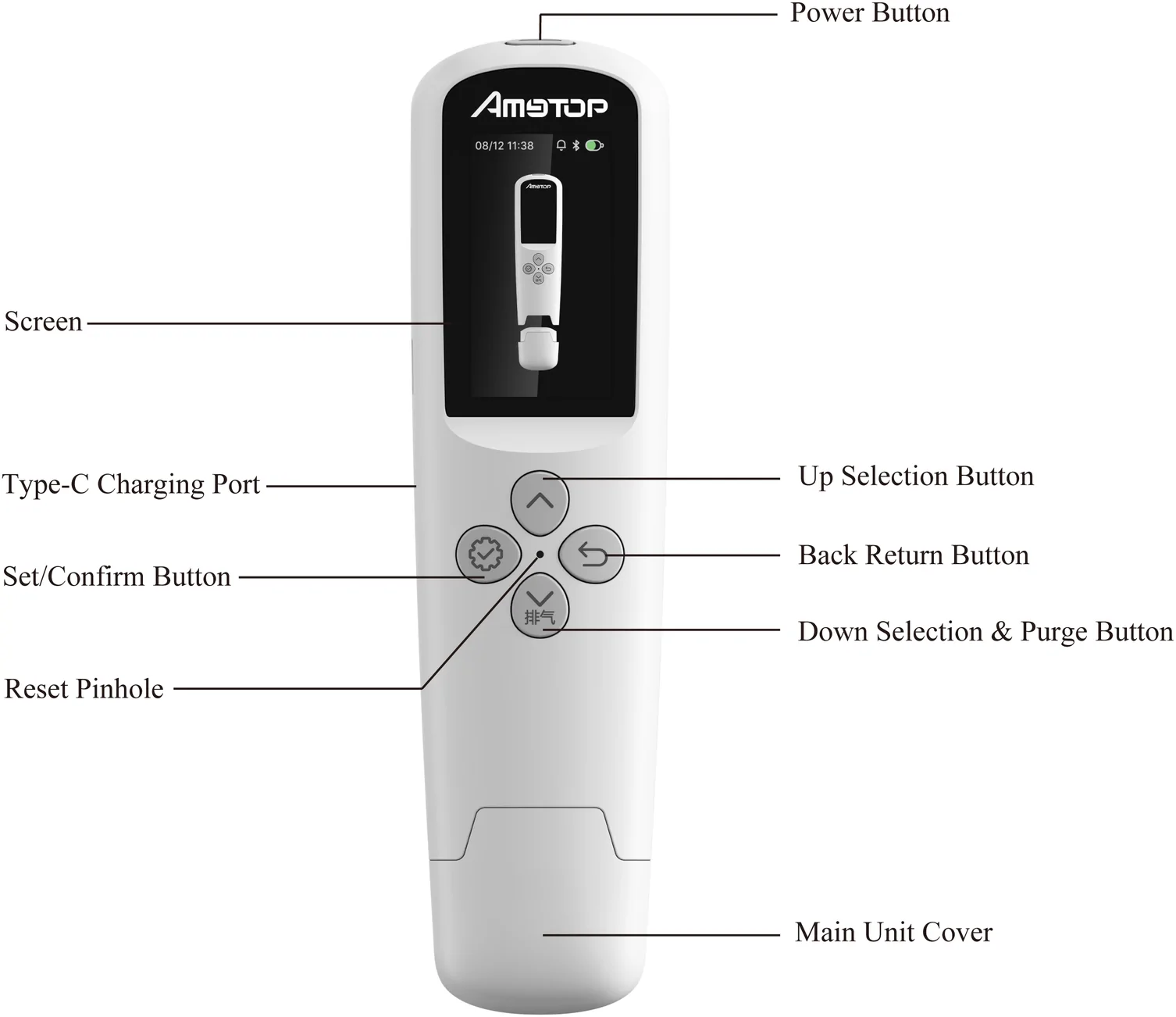
External features of Yushida. The diagram identifies the display, power, navigation controls, reset pinhole, Type-C charging port, and main-unit cover.

**Figure 2:**
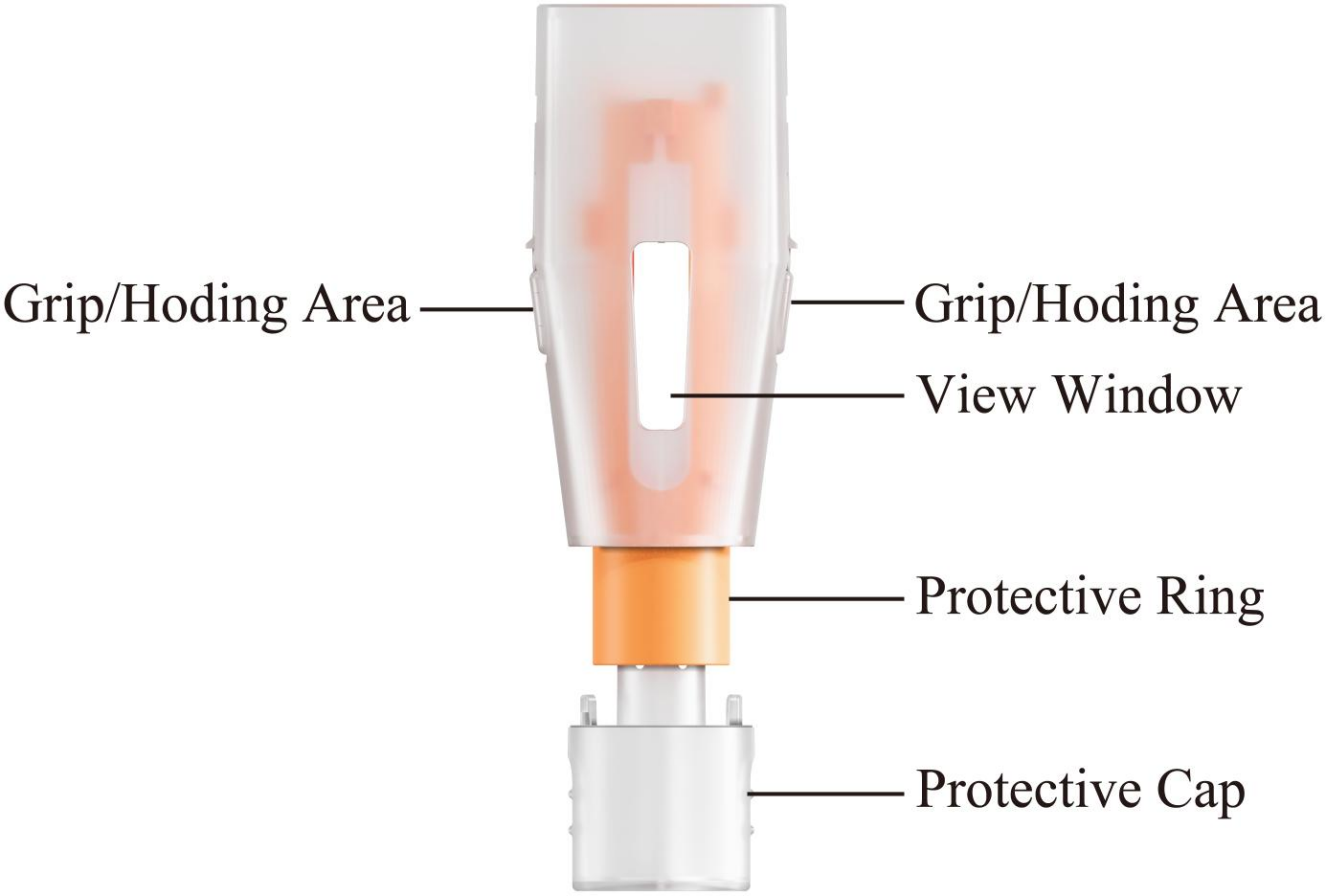
Components of the compatible pre-filled cartridge. The diagram shows the transparent grip/holding area, viewing window, protective ring, and protective cap.

The device features an ergonomic design equipped with a large liquid crystal display (Figure 1) and function buttons, allowing users to perform parameter settings, review injection history, and execute priming operations. A standard Type-C charging interface is integrated into the side of the device to support routine charging. The prefilled cartridge used in conjunction with the injection pen consists of a protective cap, a retaining ring, and a medication viewing window (Figure 2). Its concealed-needle design is intended to shield the needle from view and reduce the risk of accidental needle-stick injury. The enclosed protective shell can prevent damage caused by falls, while the disposable design contributes to ensuring the stability of the drug. Additionally, the device is compatible with several needle size, and the currently supplied cartridges use 27G and 29G penta-beveled needle designs.

### 3.2 Intelligent sensing and injection mechanism

Yushida is equipped with an intelligent recognition system, and its compatible cartridge incorporates a built-in smart chip. Upon insertion of the cartridge, the device automatically reads and verifies the drug matching information. Furthermore, the device integrates multi-functional smart assistance and safety control features throughout the injection process. Voice-guided instructions provide step-by-step operational guidance during device preparation and drug administration. The device is equipped with a proprietary intelligent sensing system (Patent No. CN 221309121 U). During use, the injection pen is positioned vertically against the injection site, and moderate pressure is applied by the user. Sensor activation initiates the internal actuation mechanism, which is designed to control injection parameters such as speed and dose delivery. According to the device specifications, the entire process can be completed within two minutes. To reduce power consumption, the device is configured to automatically shut down after a predefined period of inactivity and subsequently enter a low-power sleep mode. In the event of device malfunction, operational errors, or other abnormal conditions, the system can alert users and record relevant injection information.

### 3.3 IoT-based digital health integration

**Figure 3:**
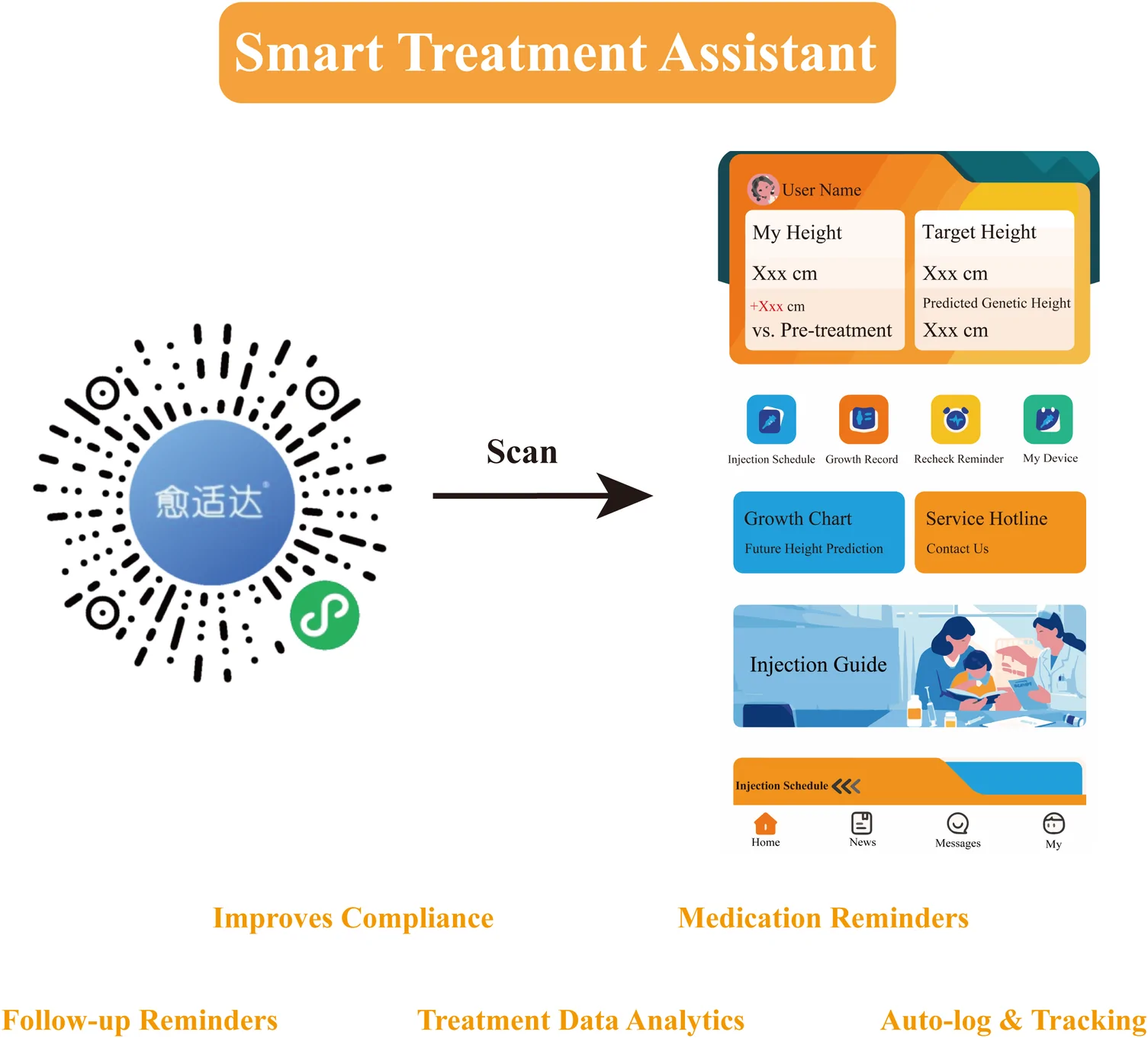
Patient-facing treatment-management interface. The WeChat mini-program can provide functions about the injection schedule, growth record, reminders, and device information.

In addition to its hardware components, Yushida incorporates an IoT-enabled data management module that connects the injection pen, which operates on an embedded system, with a dedicated mobile application (WeChat mini-program) to support digital management of the treatment process (Figure 3). Injection records can be transmitted from the device to a cloud-based data platform via Bluetooth, allowing treatment information to be stored and reviewed through the application. As shown in Figure 3, the accompanying mini-program includes functions related to data management and follow-up. These functions include medication reminders, follow-up appointment notifications, and the recording of injection schedules. For pediatric applications, particularly during growth hormone therapy, the platform additionally allows the entry and visualization of longitudinal growth curves for height and weight, which may provide useful data for continuous monitoring of growth and development. Currently, the digital platform is available only through the WeChat ecosystem and is therefore primarily applicable to users within China.

### 3.4 Data transmission and cybersecurity

**Figure 4:**
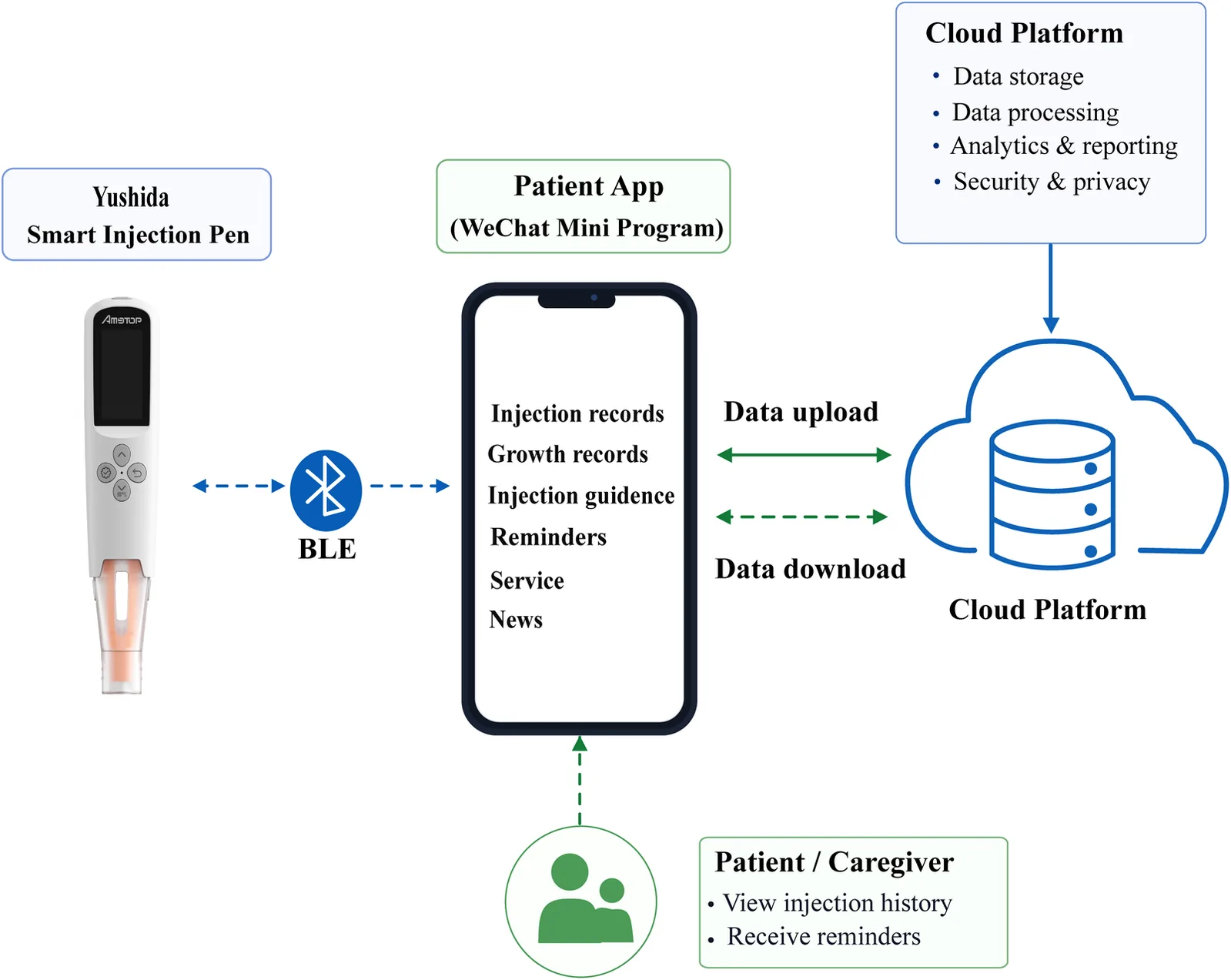
The currently fully implemented data flow architecture of the Yushida smart injection system. Injection data could be securely transmitted via BLE to the patient app and then uploaded to the cloud. BLE, bluetooth low energy.

The system transmits data via Bluetooth using AES-128 encryption. All communications between the mobile application and the cloud server are secured through HTTPS with TLS/SSL encryption to protect data confidentiality and integrity during transmission. User authentication is performed through WeChat authorization, while device authentication is established by QR code–based binding, ensuring that only authorized users and registered devices can exchange data. Before activation of the digital management platform, users or guardians are required to review and electronically consent to the privacy policy and terms of use, including data collection, transmission, and storage. Cloud services are deployed in a secured architecture with application and database isolation, HTTPS-only access, and controlled data flow to prevent unauthorized database access. The overall data transmission process is illustrated in Figure 4.

In accordance with the “Guiding Principles for the Cybersecurity Review and Registration of Medical Devices” (NMPA, No. 7, 2022) in China (27), this connected injection system incorporates cybersecurity measures throughout its lifecycle, including encrypted data transmission, identity authentication, access control, and secure cloud architecture. Independent cybersecurity assessments conducted by a qualified third-party testing organization demonstrated compliance with the applicable cybersecurity requirements, including vulnerability scanning and security verification.

## 4 Potential clinical applications and human factors considerations

### 4.1 Long-term injectable therapy and adherence management

Medication adherence is a critical determinant of therapeutic success (28, 29). Poor adherence not only compromises individual patient health and impedes the achievement of optimal treatment outcomes but also imposes a substantial health economic burden on the healthcare system (30–32). This challenge is particularly pronounced in diseases requiring long-term injectable therapies. For instance, Cutfield et al. reported that among pediatric patients receiving growth hormone therapy, those with poor adherence exhibited significantly lower annual height velocity compared to their counterparts with good adherence (33). Similarly, in the context of interferon therapy for viral hepatitis, optimal treatment efficacy is highly dependent on sustained patient adherence (34, 35). Collectively, these findings underscore the critical importance of enhancing adherence in long-term injection therapy.

Digital health technologies have increasingly been explored as potential approaches to addressing adherence fatigue associated with long-term treatment. Research has shown that patients requiring growth hormone therapy prefer smart electronic devices that integrate multiple supportive features, such as medication reminders and treatment tracking, equipped with sensors and precise cartridge verification, capable of dose presetting and adjustment, featuring automatic injection with hidden needles, and offering adjustable injection parameters (9). Additional studies have demonstrated that the use of connected devices significantly enhances patient engagement in self-management (36–38). Healthcare professionals also widely acknowledge the necessity of establishing an objective adherence monitoring system (30). In this context, Yushida integrates automated injection, electronic sensing, and IoT-based technology into a single platform intended to support treatment monitoring and adherence management. However, published evidence directly evaluating the effectiveness of the device in improving adherence or clinical outcomes remains limited, and prospective clinical studies are needed to further establish its clinical value.

### 4.2 Human factors and user experience

**Figure 5:**
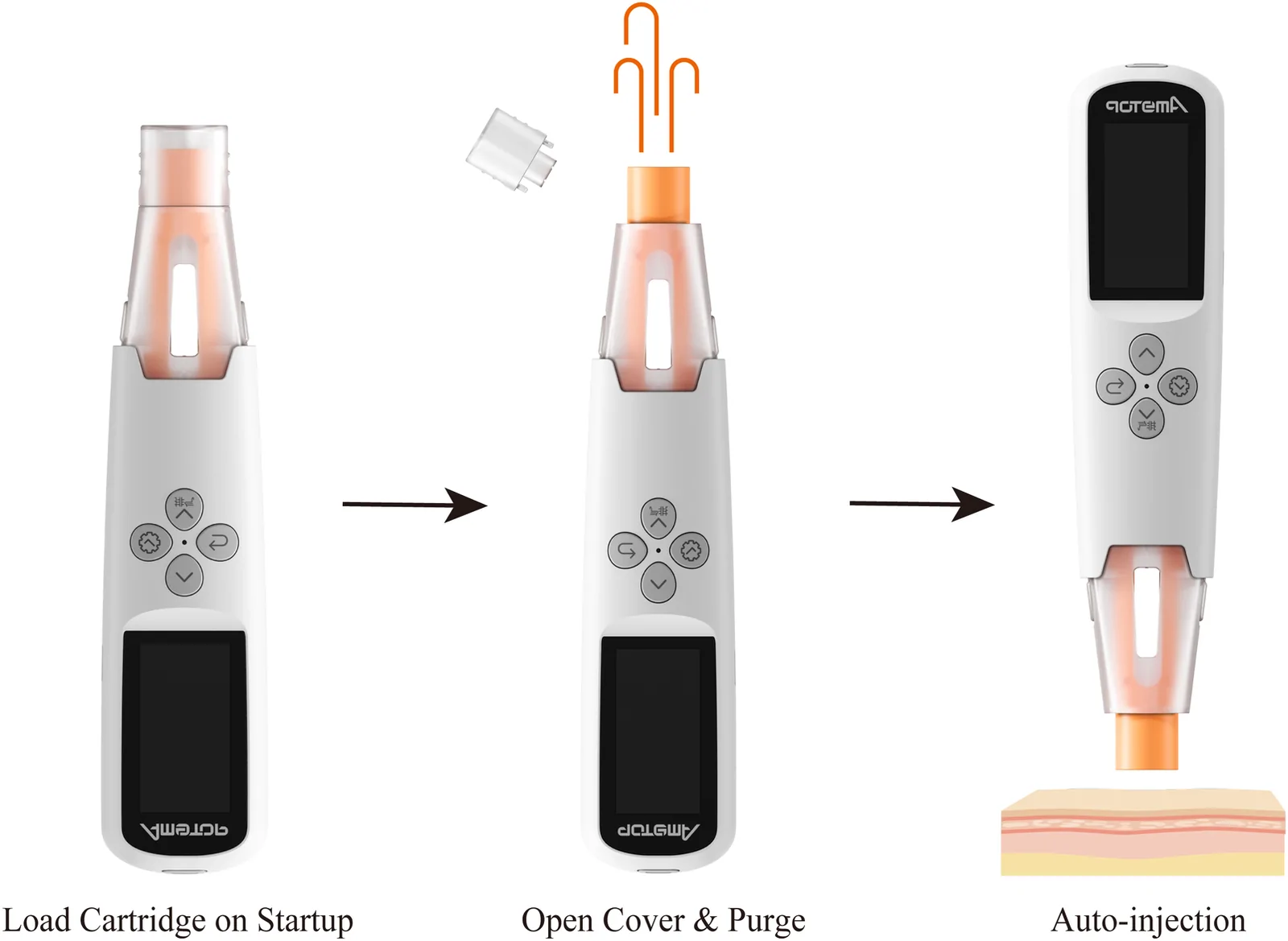
Three-step use sequence for the Yushida system: load the cartridge, open the cover and prime the device, then position it for sensor-triggered injection.

Apart from the objective functional support, the patient's subjective injection experience is also an important factor influencing adherence (9, 39, 40). Multiple studies have confirmed that patients (especially pediatric patients) tend to prefer devices that are reliable, simple, can reduce the burden of drug mixing and storage, simplify the injection preparation process, and minimize the pain during injection to the greatest extent (9, 10, 41). In response to these user's needs, the system has systematically optimized its design in three dimensions: operation process, psychological relief, and pain management:

- **Simplified injection workflow:** Yushida is designed to simplify the injection procedure through a standardized “Load-Prime-Inject” workflow (Figure 5). Leveraging the ready-to-use and disposable design of the pre-filled cartridge, along with automated priming and trigger-activated automatic injection, it eliminates cumbersome steps such as drug reconstitution, manual priming, and needle attachment. Such workflow simplification may reduce operational burden, particularly for patients requiring long-term self-administration or caregiver-assisted treatment.
- **Psychological consideration:** Needle anxiety is a well-recognized barrier to treatment adherence, especially among pediatric patients (20). The concealed needle design visually shields the needle during administration and may improve patient acceptance by reducing visual exposure to the needle. Additionally, the continuous voice guidance provides step-by-step operational instructions, which may reduce uncertainty during device operation, particularly among first-time users and caregivers. Likewise, for pediatric users who are capable of understanding and following verbal instructions, the voice-guided workflow may also facilitate more independent completion of the injection procedure.
- **Injection comfort:** Injection-related pain is influenced by multiple factors such as needle geometry, injection volume, injection site, and the physicochemical properties of the drug solution (42). Within the 26–30 G range, needle gauge does not significantly affect injection pain, whereas finer needles carry an increased risk of breakage (42, 43). Although the Yushida device is compatible with a wide range of needle gauges, it currently employs a 27G and 29G five-bevel needle design. Some studies have reported that multi-beveled needles may reduce penetration resistance compared with conventional tri-beveled needles, potentially decreasing tissue trauma during insertion (44–48). Moreover, studies have indicated that the presence of citrate in injectable formulations may be associated with increased injection site pain (49). Notably, the therapeutic agents used in conjunction with this device are formulated without citrate or preservatives, which may further reduce formulation-related irritation. Nevertheless, whether these characteristics translate into clinically meaningful improvements in patient-reported injection comfort when integrated into the system remains to be confirmed through clinical studies.

### 4.3 Digital management and clinical decision support

In the management of long-term diseases, the smart injection platform also demonstrates important value. For example, for pediatric patients requiring prolonged injectable therapies, conventional administration devices necessitate close supervision by caregivers throughout the procedure to prevent medication errors (6, 50). Digital management platforms may help reduce this workload by providing automated injection recording, medication reminders, and electronic treatment documentation (51). Through the accompanying mobile application, caregivers are able to review injection records and treatment status without relying solely on manual documentation or verbal communication. Yushida currently incorporates the above-mentioned functions, which are expected to enhance workflow efficiency and reduce communication errors.

The digital management system is expected to improve the sharing of treatment information between healthcare providers, caregivers, and patients. Compared with traditional follow-up methods that rely on patients' memory or manual recording, these objective treatment records may assist clinicians in reviewing adherence patterns and identifying deviations from prescribed treatment plans during routine follow-up. Based on these real-time and accurate injection data, doctors could provide targeted guidance and promptly adjust treatment regimens, potentially achieving precise intervention. In addition, the accompanying mini-program platform is also intended to offer digital patient support and consultation services to help users promptly resolve questions during use. The above “device-plus-service” model is anticipated to help optimize the allocation of medical resources (52, 53). However, the extent to which these digital management functions improve clinical decision-making, healthcare efficiency, or patient outcomes has not yet been evaluated specifically for Yushida and warrants further investigation in prospective clinical studies.

## 5 Current evidence and comparative perspective

In recent years, with the advancement of digital healthcare technologies, smart injection systems have gradually evolved from traditional electronic injection devices into connected digital therapy management platforms (54, 55). Currently, a variety of representative products are being used in different chronic disease areas, including the easypod® series of growth hormone injection systems developed by Merck KGaA and connected insulin pens for diabetes (such as Medtronic's InPen®) (30, 56). These systems generally possess common functionalities, including automatic injection recording, wireless data transmission, medication reminders, and remote adherence monitoring, reflecting a broader transition from drug-delivery devices to digital disease-management platforms. Likewise, although Yushida differs in some respects from these leading systems, it offers similar core features (Table 1).

**Table 1:** Summary of representative marketed smart injection systems and yushida.

Feature	Third-generation easypod®	Medtronic InPen	Yushida (current article)
Medication type	Growth hormone	Insulin	Growth hormone and interferon; expandable
Injection mode	Automatic electromechanical injection after positioning/button activation	Manual injection	Trigger-activated automatic injection
Needle exposure	Low needle exposure during injection	Exposed pen needle during manual injection	Cartridge-based low-needle-exposure design
Needle/cartridge installation	Manual needle installation required	Manual needle installation required	No separate needle installation required; cartridge is installed as a unit
Purging/priming	No separate purge step described; automatic purge not explicitly stated	Manual priming before each injection	Device-assisted automatic purge with screen and voice guidance
Operational guidance and feedback	On-screen instructions with auditory chime/sound alerts	App-based notifications and alerts; no device-level voice guidance	Screen prompts combined with step-by-step voice guidance
Connectivity/data transmission	Cellular direct to cloud (smartphone-independent)	Bluetooth-smartphone-cloud	Bluetooth-smartphone-cloud
Injection reminders	Growzen Buddy® mobile app	Companion mobile app	Mobile app
Clinician-patient data sharing	Growzen Connect® clinician portal	CareLink™ shared reports	Patient-side implementation is complete; physician-side implementation is pending.
Drug compatibility	Saizen® cartridges only	Compatible with multiple insulin brands	Independent cartridge design; theoretically adaptable to multiple formulations
Drug identification and dose confirmation	Detects and rejects incompatible Saizen cartridges	Automatically detects cartridge replacement, but users must manually verify the insulin label before each injection	Detects and rejects incompatible cartridges; uses RFID-labeled cartridges to automatically read drug information and guide dose confirmation for different specifications
Power supply	Rechargeable removable lithium battery; USB-C charging	Non-replaceable Li-Mn battery	Rechargeable device with Type-C charging port

App, application; RFID, radio frequency identification.

Among currently available connected injection systems, third-generation easypod® represents one of the most mature digital platforms (57). Compared with its predecessors, easypod® incorporates an embedded cellular communication module that enables automatic transmission of injection records directly to the Growzen™ cloud platform without requiring smartphone pairing (58). Through the Growzen Connect® portal, healthcare professionals can remotely review patients' treatment adherence. Large multinational observational studies, particularly the Easypod Connect Observational Study, have demonstrated that objectively monitored adherence is associated with improved growth outcomes in children receiving growth hormone therapy (59, 60).

In terms of system architecture, Yushida is similar to the third-generation easypod® in that it supports automatic recording of injection events, cloud-based data storage, and medication reminders. There are several differences between the two devices (Table 1). For example, Yushida employs a Bluetooth-to-smartphone-to-cloud architecture, whereas easypod® transmits data directly to the cloud using an embedded cellular communication module. The smartphone-based approach adopted by Yushida allows users and caregivers to conveniently access and review injection records, treatment progress, and reminder information through the mobile application. Moreover, this architecture may reduce hardware complexity and help with software updates but depends on the availability of compatible mobile devices and stable wireless connectivity. In contrast, the direct cellular architecture of easypod® enables greater operational independence but may involve increased hardware complexity and associated costs. In addition, because data are transmitted directly to the cloud, users generally may need to access a dedicated platform to review their records.

Similarly, in the field of diabetes, connected insulin pens (such as the Medtronic InPen®) have enabled Bluetooth-based data synchronization, automatic injection logging, and cloud-based data sharing, and real-world studies have suggested that their use is associated with improved glycemic control (51, 61). However, unlike Yushida, InPen is fundamentally a conventional manual injection pen supplemented with digital recording functions, whereas Yushida integrates automated injection, cartridge recognition, and digital management within a single electronic platform.

In addition to the established products mentioned above, emerging platforms such as Ypsomed's YpsoMate® SmartPilot™, BD's Libertas™, and Gerresheimer's Gx InMonit™ represent another direction in technological development: connecting devices to the internet via external smart modules (62–65). This modular approach offers greater flexibility for technological upgrades, while Yushida's integrated design reduces the need for additional accessories, helping to streamline operational processes.

Overall, these representative platforms illustrate a shift from standalone drug-delivery devices toward connected digital health platforms integrating automated drug delivery, adherence monitoring, and longitudinal management. Easypod® has established the clinical value of objective adherence monitoring through extensive real-world evidence, whereas InPen demonstrates the feasibility of integrating connected injection records into routine diabetes management. Yushida supports the same core digital functions (such as automatic recording, reminders, data sharing, etc.) while integrating automated injection, intelligent cartridge recognition, and IoT-enabled data management into a single platform. Importantly, its dedicated WeChat mini-program provides a localized digital ecosystem that may facilitate implementation within the Chinese healthcare setting. Although its architecture is broadly consistent with current trends in connected drug-delivery systems, further prospective studies and real-world evidence are needed to establish its long-term clinical value.

## 6 Study limitations

Several limitations should be considered when interpreting the findings of this article. First, as the system was only recently approved for clinical use in China, its real-world application has been relatively limited, and the currently published evidence remains limited owing to the relatively short time since market approval. It is worth noting that several prospective registered clinical studies involving the use of the connected injection system in combination with inpegsomatropin for pediatric growth disorders (ClinicalTrials.gov identifiers: NCT06927310, NCT07309562, and NCT07614152) are currently underway, and their findings are expected to expand clinical evidence. In addition, several prospective multicenter investigator-initiated studies, independently led by clinical investigators, are currently ongoing to systematically collect real-world data, including procedural records, adverse events, and clinical outcome measures. When more clinical evidence becomes available, comprehensive analyses will be performed to further assess the safety and effectiveness of the system and to better characterize its clinical utility and performance in routine clinical practice. Second, interoperability remains a persistent challenge across the digital health landscape. Studies have identified the lack of standardized data interfaces is one of the principal barriers to the large-scale implementation of connected healthcare platforms (66, 67). The current system operates through a WeChat Mini Program, thus it's primarily applicable to the Chinese healthcare ecosystem, without current support for interoperability with electronic health record systems outside China. Meanwhile, as a connected medical device, the system has undergone cybersecurity testing only in accordance with Chinese regulatory requirements. Cybersecurity evaluations required for regulatory approval by the U.S. Food and Drug Administration (FDA) and the European Union have not yet been performed (68–70). These limitations, to a certain extent, may present additional regulatory requirements for future deployment in international multi-center clinical studies and may affect its commercialization in the global medical market until the relevant evaluations are completed.

Third, compared with conventional mechanical injection pens, the system incorporates multiple intelligent functionalities that require additional digital infrastructure, technical support, as well as software usage learning or training. These requirements may increase implementation costs and healthcare resource utilization. The formal pharmacoeconomic evaluation of the cost-effectiveness of the Yushida system in the real world has not been published yet. Further health economic studies are needed to determine whether the higher upfront investment is offset by long-term economic benefits, such as reductions in treatment failure and associated healthcare costs resulting from improved medication adherence. Fourth, the effectiveness of connected smart injection systems depends not only on device performance but also on users' ability to interact with the accompanying digital ecosystem (71). Routine yet demanding tasks like keeping a phone charged and connected, installing software updates, or fixing broken Bluetooth pairings can become potential obstacles for older caregivers or people with limited digital skills. What's more, network dropouts, pairing failures, and data sync delays can interrupt treatment continuity in tangible ways, from missed dose records to misplaced alerts. In addition, unstable network connections, device pairing failures, or delayed data synchronization may affect the continuity of digital management. These factors may influence the long-term usability and accessibility of the system in routine clinical practice, highlighting the importance of user-centered interface design, simplified operational workflows, and appropriate patient education and technical support. Finally, this study is a technology-focused paper rather than a systematic review or meta-analysis, which may limit the comprehensiveness, transparency, and reproducibility of the literature identification and selection process. Future systematic reviews are needed to comprehensively synthesize the available evidence on smart injection systems across diverse therapeutic areas.

Overall, the Yushida system shows promising potential in terms of technological integration and usability. Nevertheless, its long-term clinical value, cost-effectiveness, and cross-regional applicability require further validation through real-world evidence. Future research and system development will focus on addressing the aforementioned limitations with the aim of establishing Yushida as an important component of the digital ecosystem for chronic disease management.

## 7 Future challenges and development perspectives

In recent years, the medical field has been accelerating its digital transformation. Digital health solutions that integrate patients, healthcare providers, and the medical system have been developing rapidly, continuously influencing disease diagnosis, treatment, and management models (66, 72). Big data, the IoT, and wearable devices, among other technologies, have created new opportunities for the full-cycle management of diseases (prevention, early screening, diagnosis, intervention, and rehabilitation) (73–75). Healthcare professionals can access complete medical records and real-time monitoring data through cloud-based systems, enabling remote consultations and treatment guidance as needed (74). Meanwhile, the management of chronic conditions such as pediatric short stature and diabetes is increasingly embracing digital transformation (76, 77). Clinical guidelines for diabetes have already recognized the value of real-time monitoring technologies and telemedicine in improving patient health outcomes (78). In light of these trends toward intelligent healthcare, it is imperative to develop digital disease management systems with connected delivery devices as their technological core.

**Figure 6:**
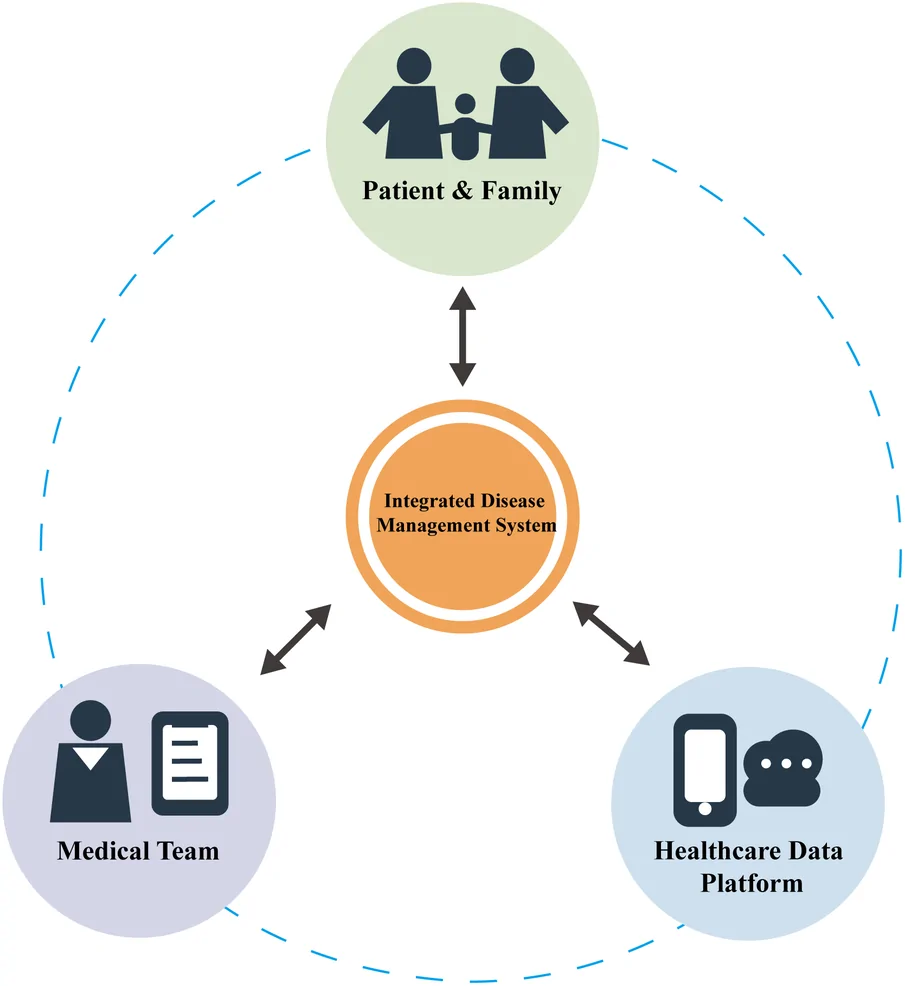
Conceptual model of the expected disease management ecosystem for the Yushida platform.

Currently, through the development of the mini-program platform, Yushida supports information connectivity at the patient end, whereas integration with the hospital information system remains ongoing. Owing to the complexity of hospital information technology systems in China and variations in implementation protocols across different institutions (79), seamless communication between patients, healthcare professionals, and medical data platforms remains technically challenging. Therefore, future development of the platform may center on improving interoperability between connected injection devices and electronic health record systems, contributing to the establishment of an integrated ecosystem encompassing devices, patients, healthcare teams, and the data cloud (Figure 6), with the ultimate goal of building a comprehensive digital health management system.

Building upon this foundation, the long-term accumulation of structured treatment records through connected injection systems has the potential to generate real-world evidence that may support future investigations into therapeutic effectiveness, dose optimization, treatment adherence, and disease progression. As evidence continues to accumulate, these data may contribute to the optimization of clinical management strategies and, where appropriate, inform future clinical research and guideline development. Additionally, artificial intelligence and machine learning algorithms have made significant advances in supporting patient care (80, 81). Numerous studies have developed machine learning-based clinical prediction models for various diseases (82–85). In the future, such smart injection platforms may integrate machine learning technologies to explore similar innovative applications. For example, machine learning algorithms could be employed to monitor dynamic changes in patients' electronic health data, predict disease trajectories, and assist in generating treatment recommendations (20, 86). Nevertheless, these applications remain exploratory and require rigorous prospective validation before routine clinical implementation.

The widespread adoption and promotion of the proposed smart injection system also face certain challenges, among which user education and awareness are particularly crucial. Studies have shown that structured health education can significantly enhance the treatment adherence of patients with chronic infectious diseases (such as hepatitis C) (87). For patients with chronic viral hepatitis receiving interferon-α therapy, disease-specific educational interventions have also demonstrated clear positive effects on self-management capacity (88). These findings suggest that maximizing the clinical benefits of this technology depends on sustained user education and outreach efforts to help target populations understand the value of IoT-enabled injection pens in long-term therapy, thereby facilitating a shift from passive treatment acceptance to proactive disease management. Furthermore, the digital management platform of the system is currently undergoing continuous iteration and optimization. Improvements will focus on operational workflows, mini-program registration and device binding processes, and the presentation of data reports, with the aim of delivering a more intuitive and streamlined user experience. These enhancements are intended to accommodate users of varying ages and educational backgrounds, lower barriers to use, and extend coverage to a broader patient population.

Currently, the Yushida system has received regulatory approval only from China's National Medical Products Administration. To enter the international market, it needs to invest a large amount of resources to meet the specific regulatory requirements of various regions. For example, the U.S. Food and Drug Administration has premarket requirements for drug–device combination products and the European Union has requirements for clinical evaluation and post-market surveillance under the Medical Device Regulation (89, 90). To improve the system's global applicability, future work will focus on planning and preparation for registration filings based on the regulatory frameworks of major target markets, including but not limited to preparing necessary technical documents, conducting targeted performance verification tests, and establishing label and instruction systems that comply with local medical infrastructure, language, and user habits, etc. Through the systematic advancement of the above international registration work, it is expected to gradually expand the application scenarios of Yushida in different medical systems and may enhance its accessibility in the global digital health ecosystem.

It is worth noting that although Yushida is currently indicated for use with specific PEGylated growth hormone and interferon formulations, its cartridge system features an independent design. Theoretically, by adapting the cartridge configuration to facilitate compatibility with different prefilled injectable formulations, stable and reliable drug delivery is expected to be achieved. This indicates that the system has the potential to evolve into a universal delivery platform for various injection formulations, and is expected to provide more flexible and convenient solutions for the treatment of different chronic diseases. Moreover, as a relatively new device, independent prospective clinical studies and multicenter real-world investigations remain necessary. Therefore, multiple clinical studies will be conducted in China in the future to evaluate Yushida's long-term safety, usability, treatment adherence, clinical effectiveness, and implementation across diverse healthcare settings, aiming to accumulate real-world evidence and further validate the comprehensive value of this integrated solution in clinical practice. Finally, because several authors are affiliated with the manufacturer of Yushida, this relationship has been transparently disclosed. Throughout the manuscript, efforts have been made to provide a balanced assessment by comparing Yushida with representative commercially available smart injection systems, discussing both its strengths and current limitations, and distinguishing device characteristics from the clinical evidence currently available. The intention is to place the Yushida system within the broader context of connected injection technologies rather than to establish the superiority of a particular device.

## 8 Conclusions

In summary, Yushida is a connected smart injection system that integrates automated drug delivery, intelligent sensing, Bluetooth-based connectivity, and digital treatment management into a single platform. This article describes its device architecture, intelligent functions, and potential applications in the long-term management of chronic diseases. Although published evidence specifically evaluating the device remains limited, its integrated design highlights the potential of connected injection technologies to support digitalized chronic disease management. Future studies are needed to further evaluate its clinical performance, real-world implementation, interoperability, and data security, thereby evaluating its role in the evolving landscape of smart drug-delivery systems.

## References

[1] Paci MM Saha T Djassemi O Wu S Chua CYX Wang J. Smart closed-loop drug delivery systems. Nat Rev Bioeng. (2025) 3(10):816–34. 10.1038/s44222-025-00328-z 42583055 PMC13458989

[2] Kaptein AA. Transjecting growth hormone: continuous nightmare or controlled nuisance? Evaluation of a new needle-free device. Patient Prefer Adherence. (2013) 7:703–8. 10.2147/ppa.s46990 23926423 PMC3728268

[3] Zeng J Ye P Wei D Li L Ma W. Tenofovir-induced osteopenia and hyperparathyroidism: a case report and literature review. Front Endocrinol. (2022) 13:1043954. 10.3389/fendo.2022.1043954 PMC9875041 36714555

[4] Majewska KA Stanisławska-Kubiak M Wiecheć K Naskręcka M Kędzia A Mojs E. Maternal anxiety in relation to growth failure and growth hormone treatment in children. Medicine (Baltimore). (2020) 99(37):e22147. 10.1097/md.0000000000022147 32925771 PMC7489751

[5] Sharma L Prakash A Medhi B. Ensuring medication and patient safety for better quality healthcare. Indian J Pharmacol. (2024) 56(6):375–8. 10.4103/ijp.ijp_109_25 39973825 PMC11913332

[6] Tyson RJ Park CC Powell JR Patterson JH Weiner D Watkins PB. Precision dosing priority criteria: drug, disease, and patient population variables. Front Pharmacol. (2020) 11:420. 10.3389/fphar.2020.00420 32390828 PMC7188913

[7] Yuen KC Amin R. Developments in administration of growth hormone treatment: focus on Norditropin® Flexpro®. Patient Prefer Adherence. (2011) 5:117–24. 10.2147/ppa.s10985 21448295 PMC3063658

[8] Ahmed SF Smith WA Blamires C. Facilitating and understanding the family’s choice of injection device for growth hormone therapy by using conjoint analysis. Arch Dis Child. (2008) 93(2):110–4. 10.1136/adc.2006.105353 17412745

[9] Dumas H Panayiotopoulos P Parker D Pongpairochana V. Understanding and meeting the needs of those using growth hormone injection devices. BMC Endocr Disord. (2006) 6:5. 10.1186/1472-6823-6-5 17034628 PMC1618831

[10] Rapaport R Saenger P Schmidt H Hasegawa Y Colle M Loche S. Validation and ease of use of a new pen device for self-administration of recombinant human growth hormone: results from a two-center usability study. Med Devices (Auckl). (2013) 6:141–6. 10.2147/mder.S50088 24039458 PMC3770891

[11] Fidotti E. A history of growth hormone injection devices. J Pediatr Endocrinol Metab. (2001) 14(5):497–501. 10.1515/jpem.2001.14.5.497 11393569

[12] Kose S Mandiracioglu A. Fear of blood/injection in healthy and unhealthy adults admitted to a teaching hospital. Int J Clin Pract. (2007) 61(3):453–7. 10.1111/j.1742-1241.2006.01150.x 17313613

[13] Schiff M Saunderson S Mountian I Hartley P. Chronic disease and self-injection: ethnographic investigations into the patient experience during treatment. Rheumatol Ther. (2017) 4(2):445–63. 10.1007/s40744-017-0080-4 28956300 PMC5696292

[14] De Pedro S Murillo M Salinas I Granada M-L Martinez M Puig-Domingo M. Variability in adherence to rhGH treatment: socioeconomic causes and effect on children’s growth. Growth Horm IGF Res. (2016) 26:32–5. 10.1016/j.ghir.2015.12.002 26774403

[15] Romacker M Schoenknecht T. Prefilled syringes: the container of choice for today’s injectables. ONDrugDelivery. (2008):4–5. Available online at: https://www.semanticscholar.org/paper/Prefilled-syringes-%3A-the-container-of-choice-for-%E2%80%99-Romacker-Schoenknecht/d6fa43c61b0de26fb4c2872a968f744b8f627fdd

[16] Romacker M Schoenknecht T Forster R. The rise of prefilled syringes from niche product to primary container of choice: a short history. ONdrugDelivery. (2008):4–5. Available online at: https://scholar.google.com/scholar?q=Romacker+M,+Schoenknecht+T,+Forster+R.+The+rise+of+prefilled+syringes+from+niche+product+to+primary+container+of+choice:+a+short+history.+ONDrugDelivery.+Prefilled+syringes:+the+container+of+choice+for+today%E2%80%99s+injectables.+2008:4%E2%80%935&hl=en&as_sdt=0,5

[17] Sacha G Rogers JA Miller RL. Pre-filled syringes: a review of the history, manufacturing and challenges. Pharm Dev Technol. (2015) 20(1):1–11. 10.3109/10837450.2014.982825 25589433

[18] Gluckman PD Cutfield WS. Evaluation of a pen injector system for growth hormone treatment. Arch Dis Child. (1991) 66(6):686–8. 10.1136/adc.66.6.686 2053787 PMC1793174

[19] Antalfy A Berman K Everitt C Alten R Latymer M Godfrey CM. The adherence and outcomes benefits of using a connected, reusable auto-injector for self-injecting biologics: a narrative review. Adv Ther. (2023) 40(11):4758–76. 10.1007/s12325-023-02671-2 37733212 PMC10567963

[20] Van Den Bemt BJF Gettings L Domańska B Bruggraber R Mountian I Kristensen LE. A portfolio of biologic self-injection devices in rheumatology: how patient involvement in device design can improve treatment experience. Drug Deliv. (2019) 26(1):384–92. 10.1080/10717544.2019.1587043 30905213 PMC6442222

[21] Liang Y Wei H Yang F Zhang H Chen L Yao H. A novel Y-shaped pegylated recombinant human growth hormone for children with growth hormone deficiency. J Clin Endocrinol Metab. (2025) 110(8):e2605–13. 10.1210/clinem/dgae816 39607675 PMC12261095

[22] Wu D Huang D Peng S Chen Y Yang F Zhang X. Efficacy and safety of entecavir, peginterferon alfa-2b and GM-CSF combination therapy: the anchor randomized controlled trial. Hepatol Int. (2025) 20:31–45. 10.1007/s12072-025-10977-2 41366186 PMC12923447

[23] Liang Y Zhang C Wei H Du H Zhang G Yang Y. The pharmacokinetic and pharmacodynamic properties and short-term outcome of a novel once-weekly PEGylated recombinant human growth hormone for children with growth hormone deficiency. Front Endocrinol. (2022) 13:922304. 10.3389/fendo.2022.922304 PMC9405430 36034448

[24] Hou FQ Yin YL Zeng LY Shang J Gong GZ Pan C. Clinical effect and safety of pegylated interferon-α-2b injection (Y shape, 40 kD) in treatment of HBeAg-positive chronic hepatitis B patients. Zhonghua Gan Zang Bing Za Zhi. (2017) 25(8):589–96. 10.3760/cma.j.issn.1007-3418.2017.08.007 29056008 PMC12770544

[25] Hao X Zeng L Liu H Lai X Yang Q Li X. Peginterferon alpha-2b combined with TDF promotes durable HBsAg loss in patients with chronic hepatitis B: a multicenter, randomized controlled, phase 3 clinical trial. AASLD Abstract. (2024).

[26] Zhou W Liao X Sun LI Zhang L Lu Q Shen S. Double-stranded polyethylene glycol modified growth hormone, preparation method and application thereof. Patent/application. (2008) Application date 2008 April 3.

[27] NMPA/CMDE. Guideline for Registration and Review of Medical Device Cybersecurity (2022 No. 7). (2022). Available online at: cncsdr.org/ggtz/ggzz/202203/t20220311_304187.html (Accessed August 4, 2026).

[28] Sonmez NIK Akici N Akici A. Medication adherence in adolescents: physiological, psychosocial, and clinical perspectives. Naunyn Schmiedebergs Arch Pharmacol. (2026) 399:12759–69. 10.1007/s00210-026-05122-1 41765948 PMC13269457

[29] Lam WY Fresco P. Medication adherence measures: an overview. BioMed Res Int. (2015) 2015:217047–12. 10.1155/2015/217047 26539470 PMC4619779

[30] Loche S Salerno M Garofalo P Cardinale GM Licenziati MR Citro G. Adherence in children with growth hormone deficiency treated with r-hGH and the easypod™ device. J Endocrinol Invest. (2016) 39(12):1419–24. 10.1007/s40618-016-0510-0 27406716 PMC5107197

[31] Usherwood T. Encouraging adherence to long-term medication. Aust Prescr. (2017) 40(4):147–50. 10.18773/austprescr.2017.050 28947853 PMC5601964

[32] Chastek B Chen CI Proudfoot C Shinde S Kuznik A Wei W. Treatment persistence and healthcare costs among patients with rheumatoid arthritis changing biologics in the USA. Adv Ther. (2017) 34(11):2422–35. 10.1007/s12325-017-0617-5 29039054 PMC5702369

[33] Cutfield WS Derraik JGB Gunn AJ Reid K Delany T Robinson E. Non-compliance with growth hormone treatment in children is common and impairs linear growth. PLoS One. (2011) 6(1):e16223. 10.1371/journal.pone.0016223 21305004 PMC3031542

[34] Prasad N Patel MR. Infection-induced kidney diseases. Front Med. (2018) 5:327. 10.3389/fmed.2018.00327 PMC6282040 30555828

[35] Lang JP Michel L Halleguen O. Treatment of affective disorder in hepatitis C. A prospective study in 50 patients. Ann Med Interne (Paris). (2002) 153(7 Suppl):2s22–30. PMID: 12518079. 12518079

[36] Bernhard G Mahler C Seidling HM Stützle M Ose D Baudendistel I. Developing a shared patient-centered, web-based medication platform for type 2 diabetes patients and their health care providers: qualitative study on user requirements. J Med Internet Res. (2018) 20(3):e105. 10.2196/jmir.8666 29588269 PMC5893891

[37] Itoh N Mishima H Yoshida Y Yoshida M Oka H Matsudaira K. Evaluation of the effect of patient education and strengthening exercise therapy using a mobile messaging app on work productivity in Japanese patients with chronic low back pain: open-label, randomized, parallel-group trial. JMIR Mhealth Uhealth. (2022) 10(5):e35867. 10.2196/35867 35576560 PMC9152720

[38] Obro LF Osther PJS Ammentorp J Pihl GT Krogh PG Handberg C. Healthcare Professionals’ experiences and perspectives of facilitating self-management support for patients with low-risk localized prostate cancer via mHealth and health coaching. Int J Environ Res Public Health. (2022) 20(1):346. 10.3390/ijerph20010346 36612667 PMC9819876

[39] Amblee A. Mode of administration of dulaglutide: implications for treatment adherence. Patient Prefer Adherence. (2016) 10:975–82. 10.2147/ppa.s82866 27330280 PMC4898439

[40] Brice R Shelley S Chaturvedi P Glah D Ashley D Hadi M. Resource use and outcomes associated with initiation of injectable therapies for patients with type 2 diabetes mellitus. Drugs Context. (2015) 4:212269–14. 10.7573/dic.212269 25657811 PMC4316811

[41] Nickman NA Haak SW Kim J. Cost minimization analysis of different growth hormone pen devices based on time-and-motion simulations. BMC Nurs. (2010) 9:6. 10.1186/1472-6955-9-6 20377905 PMC2858139

[42] Usach I Martinez R Festini T Peris JE. Subcutaneous injection of drugs: literature review of factors influencing pain sensation at the injection site. Adv Ther. (2019) 36(11):2986–96. 10.1007/s12325-019-01101-6 31587143 PMC6822791

[43] Heinemann L Nguyen T Bailey TS Hassoun A Kulzer B Oliveria T. Needle technology for insulin administration: a century of innovation. J Diabetes Sci Technol. (2023) 17(2):449–57. 10.1177/19322968211059564 34889142 PMC10012366

[44] Hirsch L Gibney M Berube J Manocchio J. Impact of a modified needle tip geometry on penetration force as well as acceptability, preference, and perceived pain in subjects with diabetes. J Diabetes Sci Technol. (2012) 6(2):328–35. 10.1177/193229681200600216 22538142 PMC3380774

[45] Vedrine L Prais W Laurent PE Raynal-Olive C Fantino M. Improving needle-point sharpness in prefillable syringes. Med Device Technol. (2003) 14(4):32–5. Available online at: https://europepmc.org/article/MED/12774577. PMID: 12774577. 12774577

[46] Coghlan J He H Schwendeman AS. Overview of Humira® biosimilars: current European landscape and future implications. J Pharm Sci. (2021) 110(4):1572–82. 10.1016/j.xphs.2021.02.003 33556387 PMC8014989

[47] Nash P Vanhoof J Hall S Arulmani U Tarzynski-Potempa R Unnebrink K. Randomized crossover comparison of injection site pain with 40 mg/0.4 or 0.8 mL formulations of Adalimumab in patients with rheumatoid arthritis. Rheumatol Ther. (2016) 3(2):257–70. 10.1007/s40744-016-0041-3 27747583 PMC5127965

[48] Pager A Combedazou A Guerrero K Tzvetkova-Chevolleau T Morel D Frolet C. User experience for manual injection of 2 mL viscous solutions is enhanced by a new prefillable syringe with a staked 8 mm ultra-thin wall needle. Expert Opin Drug Deliv. (2020) 17(10):1485–98. 10.1080/17425247.2020.1796630 32700596

[49] Lambros M Tran TH Fei Q Nicolaou M. Citric acid: a multifunctional pharmaceutical excipient. Pharmaceutics. (2022) 14(5):972. 10.3390/pharmaceutics14050972 35631557 PMC9148065

[50] NiMhurchadha S Butler K Argent R Palm K Baujat G Cormier-Daire V. Parents’ experience of administering vosoritide: a daily injectable for children with achondroplasia. Adv Ther. (2023) 40(5):2457–70. 10.1007/s12325-023-02496-z 37017912 PMC10129947

[51] Cranston I Jamdade V Liao B Newson RS. Clinical, economic, and patient-reported benefits of connected insulin pen systems: a systematic literature review. Adv Ther. (2023) 40(5):2015–37. 10.1007/s12325-023-02478-1 36928495 PMC10130105

[52] Yu L Li W Xu N Liu X Liu B Zhang Y. Patient-reported outcomes in adults with tyrosine kinase inhibitor-resistant chronic myeloid leukemia receiving olverembatinib therapy. Cancer. (2025) 131(1):e35652. 10.1002/cncr.35652 39589859 PMC11694605

[53] Chan KW Lee PW Leung CPS Chan GCW Yiu WH Cheung HM. Patients’ and clinicians’ expectations on integrative medicine services for diabetes: a focus group study. BMC Complement Med Ther. (2020) 20(1):205. 10.1186/s12906-020-02994-5 32615961 PMC7331247

[54] Yao Y Cao J Tan Z Chen K. Developing a digital health competency assessment framework for public health services: a Delphi-AHP approach. Front Public Health. (2025) 13:1634261. 10.3389/fpubh.2025.1634261 40761928 PMC12319002

[55] Badareu G Cârstina S Militaru F Siminică MI Cîrciumaru D. What is the current status of research on the impact of digitalization in medicine? A bibliometric analysis. Healthcare (Basel). (2025) 13(2):93. 10.3390/healthcare13020093 39857120 PMC11764750

[56] Bailey TS Stone JY. A novel pen-based bluetooth-enabled insulin delivery system with insulin dose tracking and advice. Expert Opin Drug Deliv. (2017) 14(5):697–703. 10.1080/17425247.2017.1313831 28359171

[57] Labarta JI Dimitri P Keiser M Koledova E Rivera-Romero O. Evaluating the usefulness and ease of use of a next-generation-connected drug delivery device for growth hormone therapy: qualitative study of health care Professionals’ perceptions. JMIR Hum Factors. (2023) 10:e46893. 10.2196/46893 37531173 PMC10433030

[58] Dimitri P Van Dommelen P Banerjee I Bellazzi R Ciaccio M De Arriba Muñoz A. Opportunities for digitally-enabled personalization and decision support for pediatric growth hormone therapy. Front Endocrinol (Lausanne). (2024) 15:1436778. 10.3389/fendo.2024.1436778 39473505 PMC11518754

[59] Polak M Bouhours-Nouet N Van Dommelen P Arnaud L Berger S Castello-Bridoux C. Adherence and growth outcomes in a large cohort of children treated with recombinant GH using a connected auto-injector. J Endocr Soc. (2025) 9(12):bvaf162. 10.1210/jendso/bvaf162 41230026 PMC12603557

[60] Rodríguez Arnao MD Rodríguez Sánchez A Díez López I Ramírez Fernández J Bermúdez de la Vega JA Yeste Fernández D. Adherence and long-term outcomes of growth hormone therapy with easypod™ in pediatric subjects: Spanish ECOS study. Endocr Connect. (2019) 8(9):1240–9. 10.1530/EC-19-0325 31484160 PMC6733364

[61] MacLeod J Vigersky RA. A review of precision insulin management with smart insulin pens: opening up the digital door to people on insulin injection therapy. J Diabetes Sci Technol. (2023) 17(2):283–9. 10.1177/19322968221134546 36326233 PMC10012386

[62] Woodley WD Yue W Morel DR Lainesse A Pettis RJ Bolick NG. Clinical evaluation of an investigational 5 mL wearable injector in healthy human subjects. Clin Transl Sci. (2021) 14(3):859–69. 10.1111/cts.12946 33278331 PMC8212760

[63] Almurashi AM Rodriguez E Garg SK. Emerging diabetes technologies: continuous glucose monitors/artificial pancreases. J Indian Inst Sci. (2023) 103:205–30. 10.1007/s41745-022-00348-3 PMC10043869 37362851

[64] Masierek M Nabrdalik K Janota O Kwiendacz H Macherski M Gumprecht J. The review of insulin pens-past, present, and look to the future. Front Endocrinol (Lausanne). (2022) 13:827484. 10.3389/fendo.2022.827484 35355552 PMC8959107

[65] Gerresheimer. Smart Autoinjector Add-on for Adherence: Gx InMonit™. Available online at: https://www.gerresheimer.com/en/customer/products-solutions/pharma/digital-solutions/digital-drug-delivery-solutions/gx-inmonit (Accessed August 4, 2026).

[66] Taralunga DD Florea BC. A blockchain-enabled framework for mHealth systems. Sensors (Basel). (2021) 21(8):2828. 10.3390/s21082828 33923842 PMC8073055

[67] Ambalavanan R Snead RS Marczika J Towett G Malioukis A Mbogori-Kairichi M. Challenges and strategies in building a foundational digital health data integration ecosystem: a systematic review and thematic synthesis. Front Health Serv. (2025) 5:1600689. 10.3389/frhs.2025.1600689 40621432 PMC12226485

[68] FDA. Cybersecurity in Medical Devices: Quality Management System Considerations and Content of Premarket Submissions. Available online at: https://www.fda.gov/regulatory-information/search-fda-guidance-documents/cybersecurity-medical-devices-quality-management-system-considerations-and-content-premarket (Accessed August 4, 2026).

[69] EU. MDCG 2019-16 Rev.1 — Guidance on Cybersecurity for medical devices. 2020. Available online at: https://health.ec.europa.eu/document/download/b23b362f-8a56-434c-922a-5b3ca4d0a7a1_en (Accessed August 4, 2026).

[70] FDA. Postmarket Management of Cybersecurity in Medical Devices. Available online at: https://www.fda.gov/regulatory-information/search-fda-guidance-documents/postmarket-management-cybersecurity-medical-devices (Accessed August 4, 2026).

[71] Azizi Z Adedinsewo D Rodriguez F Lewey J Merchant RM Brewer LC. Leveraging digital health to improve the cardiovascular health of women. Curr Cardiovasc Risk Rep. (2023) 17(11):205–14. 10.1007/s12170-023-00728-z 37868625 PMC10587029

[72] Purcărea TV. Creating the ideal patient experience. J Med Life. (2016) 9(4):380–5. PMID: 27928442. 27928442 PMC5141398

[73] Lynch C. Big data: how do your data grow? Nature. (2008) 455(7209):28–9. 10.1038/455028a 18769419

[74] Wang X Guo J Gu D Yang Y Yang X Zhu K. Tracking knowledge evolution, hotspots and future directions of emerging technologies in cancers research: a bibliometrics review. J Cancer. (2019) 10(12):2643–53. 10.7150/jca.32739 31258772 PMC6584937

[75] Scheffler M Hirt E. Wearable devices for telemedicine applications. J Telemed Telecare. (2005) 11(Suppl 1):11–4. 10.1258/1357633054461994 16035978

[76] Bae YS Park S Noh C Choi B Yi SA Kim SE. A comprehensive digital medicine platform for hypertension and diabetes care in primary care: a real-world feasibility test. Digit Health. (2025) 11:20552076251344375. 10.1177/20552076251344375 40416073 PMC12103660

[77] Tornincasa V Dixon D Le Masne Q Martin B Arnaud L Van Dommelen P. Integrated digital health solutions in the management of growth disorders in pediatric patients receiving growth hormone therapy: a retrospective analysis. Front Endocrinol. (2022) 13:882192. 10.3389/fendo.2022.882192 PMC9281444 35846336

[78] Davies MJ Aroda VR Collins BS Gabbay RA Green J Maruthur NM. Management of hyperglycaemia in type 2 diabetes, 2022. A consensus report by the American diabetes association (ADA) and the European association for the study of diabetes (EASD). Diabetologia. (2022) 65(12):1925–66. 10.1007/s00125-022-05787-2 36151309 PMC9510507

[79] Zhu T Liao P Luo L Ye HQ. Data-driven models for capacity allocation of inpatient beds in a Chinese public hospital. Comput Math Methods Med. (2020) 2020:8740457–13. 10.1155/2020/8740457 32377227 PMC7199538

[80] Jiang F Jiang Y Zhi H Dong Y Li H Ma S. Artificial intelligence in healthcare: past, present and future. Stroke Vasc Neurol. (2017) 2(4):230–43. 10.1136/svn-2017-000101 29507784 PMC5829945

[81] Tekkeşin A. Artificial intelligence in healthcare: past, present and future. Anatol J Cardiol. (2019) 22(Suppl 2):8–9. 10.14744/AnatolJCardiol.2019.28661 31670713

[82] Gossec L Guyard F Leroy D Lafargue T Seiler M Jacquemin C. Detection of flares by decrease in physical activity, collected using wearable activity trackers in rheumatoid arthritis or axial spondyloarthritis: an application of machine learning analyses in rheumatology. Arthritis Care Res (Hoboken). (2019) 71(10):1336–43. 10.1002/acr.23768 30242992

[83] Tong YT Gao GJ Chang H Wu XW Li MT. Development and economic assessment of machine learning models to predict glycosylated hemoglobin in type 2 diabetes. Front Pharmacol. (2023) 14:1216182. 10.3389/fphar.2023.1216182 37456748 PMC10347387

[84] Horton WB Barros AJ Andris RT Clark MT Moorman JR. Pathophysiologic signature of impending ICU hypoglycemia in bedside monitoring and electronic health record data: model development and external validation. Crit Care Med. (2022) 50(3):e221–30. 10.1097/ccm.0000000000005171 34166289 PMC8855943

[85] Weaver CGW Basmadjian RB Williamson T McBrien K Sajobi T Boyne D. Reporting of model performance and statistical methods in studies that use machine learning to develop clinical prediction models: protocol for a systematic review. JMIR Res Protoc. (2022) 11(3):e30956. 10.2196/30956 35238322 PMC8931652

[86] Kim MK Rouphael C McMichael J Welch N Dasarathy S. Challenges in and opportunities for electronic health record-based data analysis and interpretation. Gut Liver. (2024) 18(2):201–8. 10.5009/gnl230272 37905424 PMC10938158

[87] Lutfian L Wardika IJ Mukminin MA Zamroni AH Rizanti AP Chandra IN. Effectiveness of health education in improving treatment adherence among patients with chronic communicable diseases: a systematic review and meta-analysis. Trop Med Int Health. (2025) 30(7):598–612. 10.1111/tmi.14133 40421588 PMC12213325

[88] Asadi Noghabi AA Zandi M Mehran A Alavian SM Dehkordi AH. The effect of education on quality of life in patients under interferon therapy. Hepat Mon. (2010) 10(3):218–22. PMID 22308142. 22308142 PMC3269087

[89] U.S. Food and Drug Administration. Overview of Device Regulation [EB/OL]. Available online at: https://www.fda.gov/medical-devices/device-advice-comprehensive-regulatory-assistance/overview-device-regulation (Accessed August 4, 2026).

[90] European Parliament; Council of the European Union. Regulation (EU) 2017/745 of 5 April 2017 on medical devices: consolidated version of 9 July 2024[EB/OL]. Available online at: https://eur-lex.europa.eu/legal-content/EN/TXT/?uri=CELEX:02017R0745-20240709 (Accessed August 4, 2026).

